# The endless frontier? The recent increase of R&D productivity in pharmaceuticals

**DOI:** 10.1186/s12967-020-02313-z

**Published:** 2020-04-09

**Authors:** Fabio Pammolli, Lorenzo Righetto, Sergio Abrignani, Luca Pani, Pier Giuseppe Pelicci, Emanuele Rabosio

**Affiliations:** 1 Dipartimento di Ingegneria Gestionale, Politecnico di Milano, Via R. Lambruschini, 20156 Milano, Italy; 2 Center for Analysis, Decisions and Society, Human Technopole, Via C. Belgioioso, 20157 Milano, Italy; 3 INGM, Istituto Nazionale di Genetica Molecolare “Romeo ed Enrica Invernizzi”, Via F. Sforza, 20122 Milano, Italy; 4 Dipartimento di Scienze Cliniche e di Comunità, Università degli Studi di Milano, Via Festa del Perdono, 20122 Milano, Italy; 5 Department of Psychiatry and Behavioral Sciences, University of Miami, 1120 NW 14th St, 33136 Miami, USA; 6 Dipartimento di Scienze Biomediche, Metaboliche e Neuroscienze, Università degli Studi di Modena e Reggio Emilia, Via G. Campi, 41125 Modena, Italy; 7 VeraSci, Shannon Rd., Durham, NC 27707 USA; 8 IEO, European Institute of Oncology IRCCS, Via G. Ripamonti, 20141 Milano, Italy; 9 Dipartimento di Oncologia ed Emato-Oncologia, Università degli Studi di Milano, Via Festa del Perdono, 20122 Milano, Italy

**Keywords:** R&D productivity, Pharmaceutical innovation, Attrition rates

## Abstract

**Background:**

Studies on the early 2000s documented increasing attrition rates and duration of clinical trials, leading to a representation of a “productivity crisis” in pharmaceutical research and development (R&D). In this paper, we produce a new set of analyses for the last decade and report a recent increase of R&D productivity within the industry.

**Methods:**

We use an extensive data set on the development history of more than 50,000 projects between 1990 and 2017, which we integrate with data on sales, patents, and anagraphical information on each institution involved. We devise an indicator to quantify the novelty of each project, based on its set of mechanisms of action.

**Results:**

First, we investigate how R&D projects are allocated across therapeutic areas and find a polarization towards high uncertainty/high potential reward indications, with a strong focus on oncology. Second, we find that attrition rates have been decreasing at all stages of clinical research in recent years. In parallel, for each phase, we observe a significant reduction of time required to identify projects to be discontinued. Moreover, our analysis shows that more recent successful R&D projects are increasingly based on novel mechanisms of action and target novel indications, which are characterized by relatively small patient populations. Third, we find that the number of R&D projects on advanced therapies is also growing. Finally, we investigate the relative contribution to productivity variations of different types of institutions along the drug development process, with a specific focus on the distinction between the roles of Originators and Developers of R&D projects. We document that in the last decade Originator–Developer collaborations in which biotech companies act as Developers have been growing in importance. Moreover, we show that biotechnology companies have reached levels of productivity in project development that are equivalent to those of large pharmaceutical companies.

**Conclusions:**

Our study reports on the state of R&D productivity in the bio-pharmaceutical industry, finding several signals of an improving performance, with R&D projects becoming more targeted and novel in terms of indications and mechanisms of action.

## Background

It is no coincidence that Vannevar Bush devoted the first chapter of his *The Endless Frontier* [1] to “the war against disease”, as the life sciences and pharmaceuticals are a key area for the long term evolution of the relationships between science, innovation, economic growth and society.

Notwithstanding the persistent contribution of scientific research to pharmaceutical R&D [2–4], in the early 2000s many concerns were raised on the ongoing process of drug development, which culminated in a diffuse perception of a “productivity crisis” [5, 6]. Data showed a progressive increase of attrition rates at all stages of drug development, together with a significant increase of the time needed for the completion of clinical trials [5, 7].

Several hypotheses were introduced to explain these trends, including a gestation lag associated with the fundamental transformations of scientific knowledge bases following the “omics” revolution [5, 8, 9]. Recently, signals have started to emerge of a change of tendency: (i) the number of New Therapeutic Entities (NTE) approved by year has increased regularly [10, 11]; (ii) research in oncology has benefited from the introduction of biomarkers for the targeting of therapies [12, 13]; (iii) several innovations are shaping the process of pharmaceutical R&D, from artificial intelligence to 3D printing for drug design and production [14, 15]. In parallel, pharmaceutical companies have been rethinking the entire R&D process, implementing novel organizational solutions [16] and devoting great efforts to the early detection of non-viable drug candidates [17]. Finally, the recent upsurge of advanced therapies (e.g. CAR-T cell therapies) has been interpreted as a sign of a gestation lag of further major breakthroughs coming to an end [12, 15, 18].

Concurrently, regulatory agencies such as the US Food and Drug Administration (FDA) have worked to accelerate the drug approval process. Requests for Breakthrough Therapy Designation [19], conceived to speed up approval for drugs that exhibit outstanding performances in preclinical research, have been increasing steadily passing from an average approval rate of 33% in the first years of application (2013–2015) to 44% in more recent years (2016–2018).

In this paper, we ensure comparability of results with Pammolli et al., 2011 [5] and provide an updated and accurate picture of the current state of pharmaceutical R&D, using data on drug pipelines up to 2017. Our measures of productivity refer to the R&D process (e.g. attrition rates, phase durations), rather than to R&D expenditures [20–22]. This allows us to focus on a comprehensive data set of more than 50,000 R&D projects, whose processes have been registered with time and space signatures. Information on drug pipelines is integrated with links to an enriched patent database and to sales figures for marketed compounds. Moreover, we provide a broad classification of the indications associated with each project (“chronic”, “lethal”, “multifactorial”, “rare”) and identify the type of each institution (i.e. pharmaceutical and biotechnological companies, non-industrial institutions) involved in the R&D process either as an Originator or as a Developer of each project. Finally, we introduce two measures of novelty, respectively for project indications and mechanisms of action.

We identify the therapeutic areas that have attracted a stronger effort, and we are able to ascribe the observed changes in attrition rates to institution types, and to different configurations of Originator–Developer collaborations [23].

## Methods

### Data

Data on R&D projects has been collected from R&D Focus, a comprehensive proprietary database on pharmaceutical R&D pipelines. Data on R&D projects has been complemented establishing specific matchings with sales figures from IMS/IQVIA, and with patent data from Regpat and USPTO.

R&D Focus contains information about over 43,000 medical compounds developed until September 2018, both successful and failed. For each compound, a number of details are available. In particular, in this paper we use the following pieces of information:

- ATC codes, classifying compounds into groups on the basis of the organ on which they act and their therapeutic and chemical properties; it is a hierarchical classification envisaging five levels. We refer to the first three classification levels, defined as follows:
  - ATC1: Anatomical main group, composed of one letter. Example: *N: Nervous System*.
  - ATC2: Therapeutic subgroup, composed of two digits. Example: *N04: Anti-Parkinson Drugs*.
  - ATC3: Therapeutic/pharmacological subgroup, composed of one letter. Example: *N04B: Dopaminergic Agents*.
  Additional file 1: Table S1, lists the ATC1 codes. Navigable lists of all the ATC levels are provided by the World Health Organization^1^ and by independent online resources.^2^
- Indications, i.e., the diseases for which the compound is/will be used. To ensure compatibility with previous studies [5], each indication has been classified as rare/not rare, lethal/not lethal, chronic/not chronic, multifactorial/not multifactorial.^3^ We underline that this classification is preliminary, but can anyway be profitably employed to take into account the effects of disease types on other variables, as we will see below. Extending this classification with further categories would be an interesting future work.
- Mechanisms of action, representing the biochemical interactions and pathways through which the drug produces its effects.
- Institutions that have participated to the R&D activity, distinguishing Originators (patent owners) and Developers (institutions developing the compound).
- Codes of the patents related to the compound.
- Commercial summary, which is a description of facts and events related to the compound in natural language.

Each pair (compound, indication), which defines an R&D project, is connected with information that reconstructs its development history. The development history is the sequence of development phases that the compound has undergone until its marketing or failure for any given indication. Phases are Preclinical, Phase I, Phase II, Phase III, Registration, Marketed. Each phase is associated with date and country of reference. Only projects started in USA, EU or Japan have been taken into account. We end up with a database covering the history of 50,150 R&D projects.

IMS/IQVIA data record sales in Euros of all marketed pharmaceutical products from 2002 to 2016, in 35 countries. The database contains 202,651 products corresponding to 48,402 distinct compounds. The compound names of marketed products have been linked to the R&D compounds via text matching. In our R&D dataset we cover 2333 marketed compounds, and we have been able to connect 2123 of them with sales entries (91.0%). Globally, we identify 3584 projects, i.e., pairs (compound, indication), associated with sales figures.

We link the compounds listed in our R&D dataset with both USPTO and Regpat (EPO and PCT^4^ patents); we establish a correspondence between the compounds that we list in the R&D dataset and, respectively, 2917 USPTO patents, 3441 EPO patents and 2419 PCT patents. For 14,263 R&D projects, i.e., pairs (compound, indication), we establish an association with a patent and its assignee(s), that is, its owning institution(s). Each institution name is then disambiguated and matched to a specific institutional type. In particular, we classify each patent assignee and each developing institution according to six categories: three industrial categories (pharmaceutical, biotech and other industrial) and three non-industrial categories (university, hospital and other research centers). For 84.6% of the R&D patents we are able to classify the assignees, while for 87.7% of the R&D compounds we are able to classify all the institutions involved in the R&D process.

Additional file 1: Fig. S1, proposes a flow chart summarizing the criteria and the steps leading to the construction of the datasets employed in the experiments (R&D projects, R&D projects associated with patents, R&D projects associated with sales).

### Data processing

#### Attrition rate

The attrition rate for a given development phase in a given year is defined as the percentage of R&D projects that entered the focal phase in that year and passed to the subsequent phase within 4 years (accordingly, the maximum possible starting year in our data is 2013). If information on the subsequent phase is missing but a more advanced one is recorded, the transition is deemed accomplished without imposing time constraints.

#### Phase duration

The duration of a given development phase in a year is defined as the median time required to the R&D projects that entered the focal phase in the given year to pass to the subsequent phase. The median is computed considering transitions with duration lower than or equal to 4 years, to make a sound comparison across decades.

#### Probability of success

The probability of success of projects developed within a given ATC3 is measured by the number of projects that reach the market over the total number of projects in that ATC3. Projects started after 2013 are not taken into consideration.

#### Novelty measure

Various kinds of novelty measures have been used in the scope of drug development [24]. In this paper we introduce a more comprehensive measure and we apply it to assess, for each project, the degree of novelty of both indications and mechanisms of action:

1 $$\begin{aligned} Nov_i=\frac{1}{(n_{ind/moa,<t}+1)}\frac{N_{p,<t}}{N_{p,<t}+1} \end{aligned}$$

where $n_{ind/moa,<t}$ is the number of times an indication/mechanism of action listed in project *i* has appeared in previous projects, while $N_{p,<t}$ is the total number of previous projects. We select $\min (n_{ind/moa,<t})$ to identify the “newest” mechanism of action amongst the ones related to the focal project.

### Statistical techniques

#### Statistical tests

To assess the significance of a change in productivity measures in two different time spans, we use Wilcoxon test [25], which is a nonparametric test to detect differences in the medians of two distributions. To perform such a test on attrition rates, we compare the distributions of phase transitions, treated as a binary variable indicating failure/success for each phase occurrence, in the two time spans.

#### Changepoint detection

Changepoint detection [26] identifies the time instants (changepoints) corresponding to abrupt changes in a function. Identifying the changepoints divides the function into sections. In particular, we split the attrition rate in correspondence of the years where the regression line changes the most. This is obtained by finding the sections of the function such that the sum of the residual errors of the regressions in each section is minimized. Note that adding more changepoints keeps reducing the value of the residual error, leading to overfitting. To avoid this problem, the error metric needs to envisage also a term penalizing high numbers of changepoints. Let $x_1, \ldots , x_n$ be the points of the function that we are studying, and let $f^{p,q}$ be the regression line approximating the function between the time instants *p* and *q* ($p<q$). The changepoint detection procedure finds the *K* time instants $k_1, \ldots , k_{K}$ minimizing the following metric:

2 $$\begin{aligned} J(K) = \sum _{r=0}^{K}{\sum _{i=k_r}^{k_{r+1}-1}{\left(x_i - f_i^{k_r,k_{r+1}-1}\right)^2}} + \beta K \end{aligned}$$

where in this formula $k_0$ represents time instant 1 and $k_{K+1}$ represents the last time instant (*n*). The internal summation describes the residual error of the regression between the time instants $k_r$ and $(k_{r+1}-1)$. The term $\beta K$, where $\beta$ is a parameter to be set, penalizes the addition of new changepoints. It can be easily shown that a new changepoint is rejected if it does not bring an improvement to the residual error of the regression at least equal to $\beta$. In this work the threshold $\beta$ has been set to twice the variance of the function, meaning that we stop adding changepoints when the subsequent new one would increase the $R^2$ determination coefficient of the regression of less than 2/*n*.

#### Regression with dummy variables

We model a set of response variables in a regression framework:

- Phase-by-phase transition: binary variable identifying the successful passage from the focal phase to the next one;
- Sales: logarithm of the sum of sales of the focal drug.

Our main explanatory variable is the binary variable identifying the type of Originator–Developer (O–D) relationship under study. For each project, we define the Originator(s) according to the assignee(s) of the related patent(s), and the Developers according to the developing institution listed at each stage of the R&D process. We define O–D relationships according to the presence of at least 1 assignee or developer in one of the different institutional types. We treat the “university”, “hospital” and “other research” classifications as “non-industrial”. Also, we define as the baseline O–D relationship the one that has a pharmaceutical company acting both as Originator and Developer. Then, we study five possible O–D relations: non-industrial (O) and pharmaceutical (D); biotech (O) and pharmaceutical (D); non-industrial (O) and non-industrial (D); non-industrial (O) and biotech (D); biotech (O) and biotech (O).

In addition, we use a few dummy variables to control for fixed effects characterizing the focal project: the starting year, the indication and the classification of the indication. Please notice that the four indication classes that we have indicated above (i.e. “lethal”, “chronic”, “rare”, “multifactorial”) are overlapping, and therefore multiple fixed effects related to the indication type may be relevant for a given R&D project.

In synthesis, the regression model for the generic response variable *X* can be written as:

3 $$\begin{aligned} X & = \alpha \, OD + \sum _{t=1}^{N_y}\,\beta _t year_t + \sum _{i=1}^{N_i}\,\iota _i\, indication_i \nonumber \\ &+ \kappa \, chronic + \lambda \, lethal + \rho \, rare + \mu \, multi-factorial \end{aligned}$$

where OD is the binary variable classifying each project by either a relevant project according to the O–D relationship under study, or a baseline project (pharmaceutical as originator and developer both).

## Results

### The evolution of R&D productivity in pharmaceuticals

**Fig. 1:**
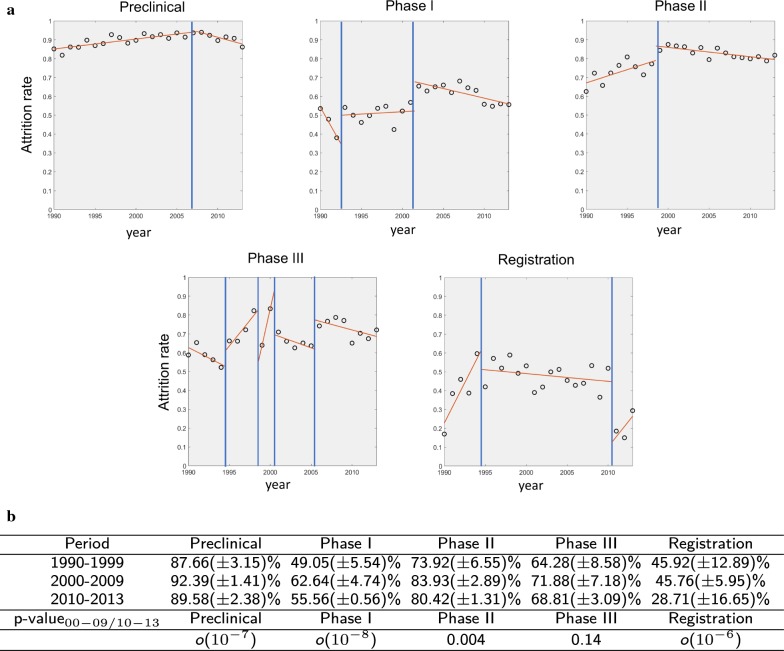
**a** Time evolution of attrition rates at different stages of drug development. Black circles: data; red solid lines: linear regression in the corresponding time window; blue vertical solid line: changepoint. In a given year, the attrition rate for each development phase is defined as the percentage of projects that started the phase in that year and failed to pass to the subsequent phase within 4 years (accordingly, 2013 is the last year we do consider).** b** Average (± standard deviation) yearly phase-by-phase attrition rates in three different time intervals (1990–1999, 2000–2009, 2010–2013). We also report the significance level of a Wilcoxon rank sum test [25] on the difference of attrition rates in 2000–2009 and 2010–2013

We identify an R&D project as a specific indication-compound association, and select projects started in either the US, Europe or Japan since 1990. We first focus on phase-by-phase attrition rates (Fig. 1a). At each stage of development, we define a success when we observe a transition to the next stage within 4 years, or, in case of missing data, to any other subsequent phase, without time constraint. As a consequence, in our analysis of attrition rates we study projects which entered any phase of development by 2013, while we consider data until 2017 to detect phase transitions. We use changepoint detection analysis [26] to pinpoint the most relevant shifts in regression slopes in the data. We have found that attrition rates in clinical phases have been declining in recent years, though they have remained above the values observed in 1990–1999. We also observe a reduction of attrition rates in preclinical research. To portray a comprehensive picture of recent trends, we show in Fig. 1b the average values of phase-by-phase attrition rates in the three decades under study. Tests on phase transitions for phases started in 2000–2009 and 2010–2013 (ibidem) show that the observed decreases are statistically significant for all phases, except for Phase III (see "Methods" for details). Attrition rates in late-stages clinical trials (i.e. Phase II and III) remain quite high (Fig. 1a, b). Market launches (i.e. projects that are registered by a regulatory agency and marketed, see the Registration panel in Fig. 1a) have increased steadily.

**Fig. 2:**
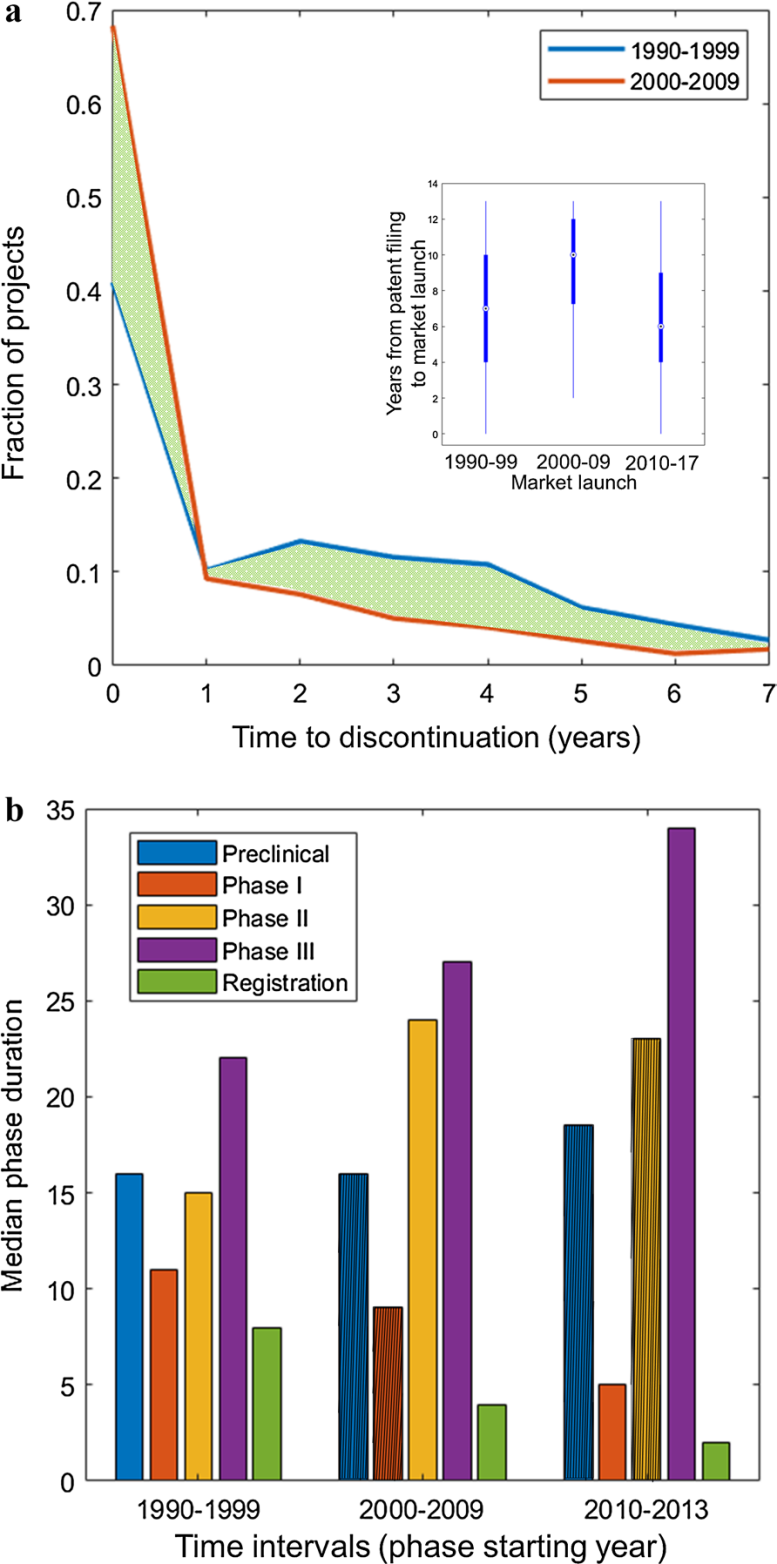
**a** Time needed for project discontinuation; 1990–1999 (blue), 2000–2009 (red). We highlight in green the area between the two curves. We show the fraction of projects that are discontinued after *x* years from the start of preclinical research. The distribution accounts for a maximum discontinuation time of 8 years, so we focus on projects started before 2010. Inset: boxplot of the time interval between patent filing and market launch years, based on the year of market launch, in three different time intervals (1990–1999, 2000–2009, 2010–2017). **b** Median phase duration per each phase of the drug development process, in three different time intervals (phases started in 1990–1999, 2000–2009, 2010–2013). The duration of a development phase in a year is defined as the median time required to the projects that started the focal phase in the given year to pass to the subsequent phase. The median duration is computed considering only transitions with duration lower than or equal to 4 years, to make a sound comparison across decades. When the median of a phase duration is not significantly different from that of the previous decade, the corresponding value is barred

**Fig. 3:**
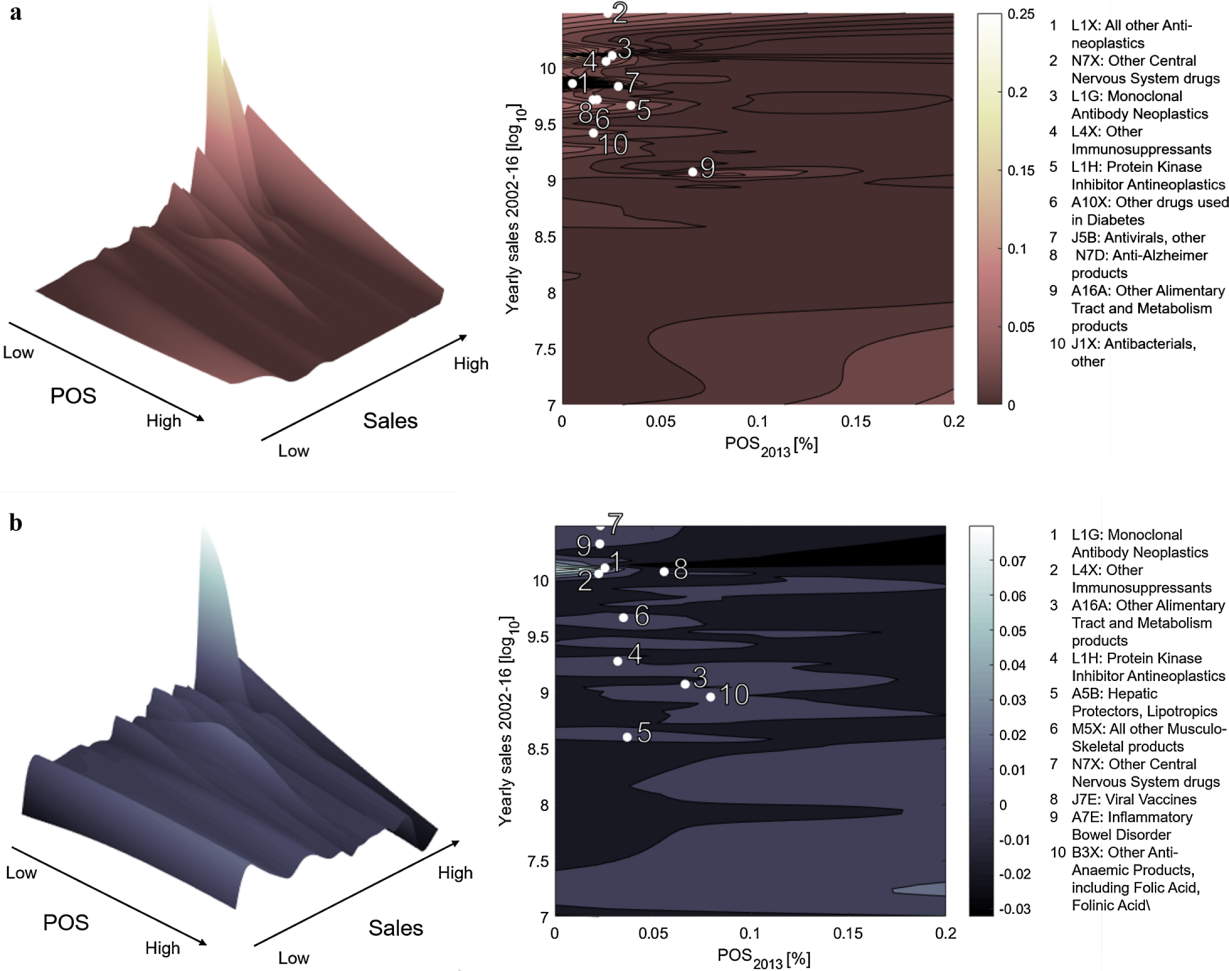
Distribution of R&D projects, by probability of success and size of the market. In each panel, the probability of success (POS) is shown on the x axis and the logarithm of potential sales (yearly average computed in 2002–2016) on the y axis. A contour plot and a three-dimensional view of the same distribution are shown. In the contour plot we highlight the top 10 ATC3 classes by the focal metric being shown on the vertical axis. These are listed besides the contour plots. **a** The vertical axis shows the percentage distribution of research and development (R&D) projects by POS and sales level. The distribution of R&D efforts is concentrated in the upper left hand corner of the plot (indicating high sales and low POS). **b** The vertical axis shows the share variation between 2002–2009 and 2010–2017, again as a function of POS and sales. Positive values (peaks in the plot) represent areas in which the research efforts have increased from 2002–2009 to 2010–2017, whereas negative values (holes in the plot) correspond to a reduction of research intensity

As a first attempt to identify drivers of decreasing attrition rates, we compute the relative performance of R&D projects targeting different therapeutic areas. To this end, we classify the projects according to their corresponding first-level ATC class. Additional file 1: Table S2 reports the values involved in this computation (phase-by-phase attrition rates, project share, significance level of the observed changes). Results provide a few relevant insights. First, at early stages of development (Preclinical and Phase I) attrition rates have been decreasing quite ubiquitously, but significant reductions (Wilcoxon test [25] at 0.05 significance level) are to be ascribed to cancer research (ATC class L: Antineoplastic and Immunomodulating Agents). Considering its share in the data, this result makes oncological research of utmost importance in the overall attrition rate reduction that we do observe for early stages of pharmaceutical R&D. Then, at later stages of the drug development process, significant reductions occur in class J (General Anti-Infectives Systemic), which also shows a statistically significant improvement in Phase III, in class B (Blood and Blood Forming Organs), in class C (Cardiovascular System) and in class P (Parasitology). When we move to consider ATC classes that provided a negative contribution to the decrease of phase-by-phase attrition rates, only a few of the observed results are statistically significant, with the notable exception of increase of Phase III attrition rates for class N (Nervous System), confirming the difficulty of research in mental/brain diseases [27].

In order to get some further insights on the observed results, we focus on specific subsets of R&D projects: two important sets of biologics, i.e. advanced therapeutics (cell and gene therapies) and monoclonal antibodies, and the R&D projects related to Alzheimer’s disease. Biologics in fact are experiencing a remarkable growth in sales: according to EvaluatePharma [28], in 2020 sales of biological compounds are expected to increase by 50 billion USD. Finally, R&D projects on Alzheimer’s disease represent a large class of neurological R&D projects [29]. In Additional file 1: Table S3, we show attrition rates in 2000–2009 and 2010–2013 for advanced therapies and monoclonal antibodies, while in Additional file 1: Table S4 we show attrition rates for Alzheimer’s disease for the same periods, including also a focus on the projects connected to the amyloid hypothesis,^5^ which we were able to identify in our data. As per advanced therapies, the significant decrease of attrition rates in the early phases of development is confirmed, with a very remarkable reduction for Phase I; please notice that the development of these therapies has been growing in recent years and so we do not observe any project passing Phase III until 2013, while our data contain eight projects reaching the market in 2014–2017. For monoclonal antibodies we record a significant decrease of attrition rate for the Preclinical phase. Regarding Alzheimer’s disease, Additional file 1: Table S4 highlights the absence of significant improvement in attrition rates; in particular, our data do not record any R&D projects passing Phase III. The further focus on the projects related to the amyloid hypothesis, accounting for about 50% of the Alzheimer’s projects, shows a similar pattern.

We now analyze the duration of R&D activities at different stages of the drug development process. First, we measure the time needed to identify non-viable R&D projects (Fig. 2a). Interestingly, $\simeq$ 70% of projects that had started between 2000 and 2009 were terminated in the year they entered preclinical research, with a $\simeq$ 20% increase with respect to the previous decade. For successful projects, we measure the time lag from date of patent to date of market launch. In the inset of Fig. 2a we show the distribution of the time lag between patent filing and market launch of successful projects in the three decades under study. Interestingly, despite the increase observed for projects started in the 1990s and in the 2000s, this measure has decreased, showing that the development of at least a fraction of the projects has become faster in recent years.

To track the evolution of phase durations, we compute the time needed to progress along the pipeline in the different decades of observation (to ensure comparability of projects in different decades we imposed a constraint of 4 years (48 months) as the maximum observable duration for a given phase). As shown in Fig. 2b, the time needed to complete preclinical research has been slightly increasing, but we did not find significant differences between decades (Wilcoxon test [25] at 0.05 significance level). In Phase I of clinical research, projects progression has become significantly faster in the latest decade. The duration of Phase II saw a significant increase in 2000–2009, but then this trend has halted. The duration of Phase III has increased progressively and significantly remaining the longest, due to the complexity of inherent activities (regulatory requirements, increasing patient sample size, multi-center logistics [6]).

### Finding the niche

Evidences presented in the previous section documented that attrition rates have been decreasing in recent years. We now move to investigate which therapeutic areas research has focused on.

To this end, similarly to Pammolli et al. [5], we partition projects under study based on their ATC, identifying their main therapeutic areas at the 3-digit hierarchical level (ATC3). In Fig. 3 we show how projects are distributed across therapeutic areas, as a function of the corresponding probability of success (POS; i.e. how many projects have reached the market from the preclinical phase, overall) up to 2013 and of the yearly average sales between 2002 and 2016. In general, results show that high uncertainty/high potential reward projects (i.e. low POS and high yearly sales) continue to polarize Research and Development efforts (Fig. 3a) [5]. Expectedly, projects in therapeutic areas with higher revenues and higher attrition rates have experienced the highest share increase between 2002–2009 and 2010–2017 (Fig. 3b). In particular, monoclonal antibody neoplastics (L1G) and immunosuppressants (L4X) have increased their share, while the still prevailing class L1X (antineoplastic and immunomodulating agents) has remained constant.

**Fig. 4:**
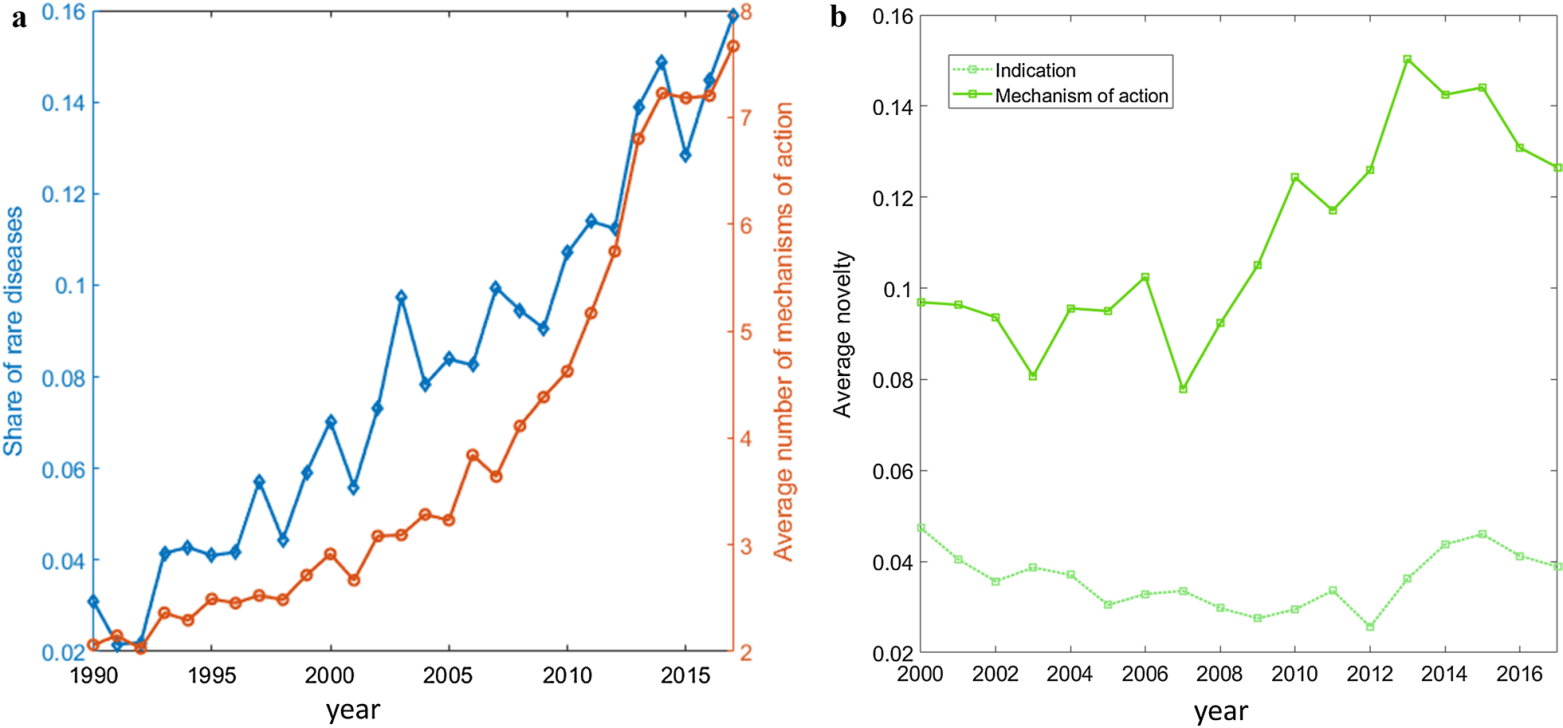
Share of rare indications, number of mechanisms of action, and novelty of indications and mechanisms of action. **a** Evolution in time of the share of projects targeting rare diseases (i.e. having a prevalence of fewer than 200,000 affected individuals in the US) and of the average number of mechanisms of action per project, between 1990 and 2017, by project starting year (i.e. the year the focal project entered preclinical research). **b** Evolution in time of median novelty of indication/mechanism of action per project, between 2000 and 2017, by project starting year

The concentration of projects in oncology has become even more apparent in the last decade: (i) 4 out the top 5 ATC3 classes, ranked by their overall share in projects ongoing between 2000 and 2017, are related to oncology (L); (ii) more than 40% of ongoing studies, currently listed on ClinicalTrials.gov^6^ are oncology-related (see Additional file 1: Table S5). Other relevant fields that showed up in rankings include degenerative diseases of the central nervous system (N7X), with specific reference to Alzheimer’s disease (N7D), another area in which unmet medical need is high [32, 33]. Interestingly, coherently with results presented in the previous section, while projects in cancer research have improved their attrition rates after 2010, the performance of projects in class N has worsened in most cases (see Additional file 1: Table S2). In fact, out of 86 projects on Alzheimer’s disease in the last 10 years, only one has received approval [15].

The focus on R&D projects of high complexity in relatively unexplored areas is also witnessed by the fact that orphan drugs indications and approval have increased in recent years [34]. The number of yearly NME approvals for orphan drugs has more than doubled from 2000–2009 to 2010–2017, while drug repositioning approvals towards rare indications have tripled in the same period [34]. We use a manual classification of indications to retrieve the share of rare diseases (defined as having a prevalence of $\le$ 200,000 affected individuals in the US) by year of project start (Fig. 4a). In the observation period, this share has increased from 3$\%$ in 1990 to about 16$\%$ in 2017.

The general tendency towards development of drugs on orphan indications and treatments that are more and more specific and target relatively small subpopulations seems to act as a factor of increasing difficulty of projects. It has been observed, for instance, that orphan drug development takes, on average, 2.3 years longer [35]. This is due, for instance, to the recruiting challenges associated with smaller and geographically dispersed patient populations, and to the scarcity of available animal models and biomarkers. An additional factor of complexity might be the increasing relevance of multifunctional drugs, which have emerged, in opposition to single-target drugs, as a new approach to treatment [36–38].

We then consider the average number of mechanisms of action per drug, by starting year of the project (Fig. 4a), observing a clear positive trend with a pronounced increase after 2010. This trend is confirmed also for the individual ATC1 classes, as shown in Additional file 1: Fig. S2. Between 1990 and 2017, the average number of mechanisms of action per drug has nearly tripled. This result may reflect a general improvement of drug efficacy, as they act on multiple targets, while it also reflects the increasing difficulty of drug design [37].

The evidences that we have presented so far might seem to lead to apparently contradictory conclusions: on the one hand, we found that research is focusing on difficult and risky areas like oncology and rare diseases; on the other hand, we observed a recent increase in productivity. A few explanations for our results can be introduced. As per cancer drugs, recent reports [11, 14] show findings similar to ours and predict even greater sales and market share, due to the combination between high medical need and advances in the relevant science basis [6]. Advances in scientific knowledge bases sustaining R&D activities in oncology are worth mentioning here. In fact, our data show that advanced therapies (i.e. cell or gene therapies) are mostly focused on oncology (Additional file 1: Fig. S3), and that projects on advanced therapies have been on a steep rise in the last few years (Additional file 1: Fig. S4) [15]. Also, the rising importance of anti-cancer antibodies (class L1G) could be a factor of simplification of drug preparation for preclinical test and clinical trials, as the efficiency of monoclonal antibody production has improved significantly in recent times [39].

As per orphan indications, FDA data [40] show that they cover a majority share in fast-track programs, while projects in these areas are affected less by the “better than the Beatles” problem described by Scannell et al. [6]. In fact, in Additional file 1: Table S6 we show that the average phase-by-phase attrition rates have been declining in the subset of projects focusing on rare indications, with the notable exception of Phase III, in which trials set-up is known to be more demanding because of the small size of target population [35].

To complete the analysis, we investigate the degree of novelty associated with each research project. For each R&D project, we measure the novelty of indications and mechanisms of action. To this end, we devise an indicator that counts the number of times a given indication/mechanism of action listed in the focal project appeared in earlier projects, taking into account the total number of previous projects (see "Methods" for details). Interestingly, the median value of both these measures has been increasing in recent years (Fig. 4b). In other words, research has tended to focus on novel indications and mechanisms of action. Recent reports [15] show, in fact, that 34% of mechanisms of action in FDA-approved drugs in 2018 are first-in-class (i.e. they were different from those of existing therapies). To gain insights into the relationships between novelty and market launches, we divide our dataset in successful (i.e. marketed) and failed projects. We have found a significantly higher median novelty of successful projects (0.083 vs 0.015; a Wilcoxon test rejects the null hypothesis that the two distributions have the same median with $\text {p}<<0.01$). In other words, an increasing fraction of marketed drugs tend to be based on novel mechanisms of action and target novel indications.

### The division of innovative labor

As shown in [5, 41, 42], the contribution of different institutions (pharmaceutical and biotech companies, non-industrial institutions) to R&D performance might differ significantly. In this section, we first investigate the role of different institutional categories as *Developers* of R&D projects. Then, we study the Originator–Developer contractual relationships, where the *Originator* of a drug project is defined as the institution that holds the relevant patent and is assumed to have started the R&D project.

We distinguish pharmaceutical companies, biotech companies, universities, hospitals, other non-industrial research centers. Overall, we cover a subset of the projects (see the "Data" section) that shows statistics comparable to the whole sample (Additional file 1: Table S7). In Table 1 we list attrition rates for each institution type and the corresponding share of projects developed in the two periods. Here we focus on Developers, while in the last part of this section we concentrate on Originators. We measure the contribution of institution type *i* to the variation in attrition rates in phase *p* for phases started in 2000–2009 and 2010–2013 using the formula $\delta _{ip}=(\Delta AR_{ip}\cdot Sh_{i})*100/\Delta AR_{tot,p}$, where $\Delta AR_{ip}$ is the variation observed in attrition rates in *p* in the projects developed by *i*, $Sh_i$ is the share of phases belonging to projects developed by *i* in 2010–2013, and $\Delta AR_{tot,p}$ is the total attrition rate observed for phase *p* for all projects for which institution classification was available. Strikingly, results show (Additional file 1: Fig. S5) that a relevant fraction of the observed reduction of the attrition rates are to be ascribed to projects developed by biotechnological companies, with significant attrition rate decreases (Wilcoxon test [25] at 0.05 significance level) in all phases (except for Registration). The contribution of pharmaceutical companies to the total attrition rate changes tends to be high due to to the large share of projects in which they act as Developers (see e.g. in Phase I), but no significant changes are reported (except for Registration). Finally, non-industrial institutions acting as Developers experience a reduction of their attrition rates in Phase II and Registration.

We now focus on the Originator–Developer relationships. While division of innovative labor and R&D alliances have become increasingly important within the industry [43], academic and non-industrial institutions have been advocated as pivotal in driving early development of candidate drugs [44], while influence of interfirm and public/private knowledge transfer on R&D productivity has been underlined [45, 46]. We study the effect of different Originator–Developer (O–D) relationships on attrition rates and sales for marketed products (i.e. the logarithm of composite sales for 2002–2016). In general, we identify an O–D relationship for each of 4860 R&D projects (1863 in the decade 1990–1999, 2997 in 2000–2013). In Additional file 1: Table S8 we show a full count of these projects by the corresponding relative O–D relationship. We have found that since the year 2000 biotechnological companies have increased their role both as Originators and Developers, while pharmaceutical companies are now less dominant as Developers than they were in the past. This trend seems to be confirmed by recent reports [15]. In Table 2 we show the results of the regressions of two response variables, phase-by-phase transition rates and sales, accounting for different O–D relationships against the baseline (i.e. a pharmaceutical company being both Originator and Developer; the complete results can be found in Additional file 1: Table S9). We run the regression for data before and after 2000, taken as a reference year. We consider fixed effects of time and a broad proxy for project difficulty, based on the classification of the targeted indication as “chronic”, “lethal”, “rare”, “multi-factorial”.

The transition rate results presented in Table 2 lead to two main conclusions. First, no O–D configuration seems to outperform the baseline. This result, while not surprising, confirms that large pharmaceutical companies have kept strong development capabilities, while they have continued to improve in discovery and preclinical research, also through acquisitions of small, research intensive, biotechnology companies [4, 23, 43]. However, biotech firms have increased the share of R&D projects in which they act as Developers, and their performance has improved over time, converging to the benchmark provided by the baseline. This result is important, because it is showing that the transition of some biotechnology companies from being oriented mostly to discovery to becoming integrated pharmaceutical companies has been providing a positive contribution to the recent recovery of R&D productivity in pharmaceuticals.

When we consider the size of the market, we see that since the early 2000s biotech firms and non-industrial institutions after 2000 have acted as Developers of R&D projects leading to smaller markets. This result is coherent with the higher share of projects focusing on an indication specified as “rare” (last columns of Table 2).

## Concluding discussion

Our analyses in this paper revealed significant improvements in different features of R&D productivity in pharmaceuticals. Attrition rates at all stages of drug development have decreased. Our findings are statistically significant, except for Phase III, due to the low number of observations after 2013. The recent decrease of attrition rates in preclinical research is a piece of evidence that will deserve further monitoring. Research on CNS has continued to experience the highest attrition rates. We found that pharmaceutical R&D has continued to focus on therapeutic indications where medical need is high (i.e. oncology and degenerative diseases of the CNS). These increasing efforts correspond to high uncertainty and high potential reward projects. Interestingly enough, we found evidence that the time to discontinuation of non-viable projects has tended to decrease.

As a possible driver of decreasing attrition rates at all stages of pharmaceutical R&D we mention the higher reliance on validation of drug targets in preclinical research, in terms of their role in the disease and their toxicity. Indeed, the extensive genetic validation of drug targets has become more widely embraced in different therapeutic areas [47, 48] and it has been shown to improve the chances of passing through clinical stages [49]. Better selection of patient subsets for clinical trials via “stratification” based on biomarkers [50] is a possible factor of improvement of success rates. “Precision” diagnostic assays are increasingly used as clinical endpoints [51], contributing to strengthen selection capabilities in drug development. Finally, the higher number of monoclonal antibodies as new candidate drugs has positively affected both preclinical development and clinical grade batch preparation [52].

We found that many of the detected improvements are widespread across projects in different therapeutic areas and at different stages of development, except for Phase III, in which performances show a higher variability and the impact of molecular stratification of patients seems to be still in its infancy. R&D projects on different types of cancer have experienced significant attrition rate decreases in early stages of development (Preclinical and Phase I) while improvements at later stages (II and III) have been driven more by anti-infective drugs.

The low productivity in CNS research can be explained by several motivations: e.g., patient heterogeneity, complexity of neurodegenerative diseases that often involve multiple molecular targets, the relatively low predictive validity of experimental animal models, the relative lack of established clinical biomarkers [27, 53]. To improve this situation, changes are needed in both therapeutic research and regulatory policies, and specific programs and initiatives to promote such changes are being undertaken [54, 55]. At present, R&D productivity in CNS is still lagging behind.

Moreover, our analyses showed that the number of mechanisms of action in drug projects has grown over time, and that the novelty of mechanisms of action and indications has increased. New drugs are increasingly based on novel mechanisms of action. The rise in the number and novelty of mechanisms of action and indications that we discovered for recent projects and the increasing focus on high uncertainty and high potential reward projects shows that new research trajectories are opening up. We found, though, that phase duration at late stages of drug development is increasing, particularly in Phase III, pointing at requirements in terms of trial organization and outcomes. Our findings documented that increasing numbers of candidate drugs tend to target multiple (and novel) mechanisms of action, following improvements in the understanding of the genetic, molecular and cellular bases of diseases. Though this paradigm shift may result into the generation of more efficacious drugs, it might also affect the length of the process of drug design. In fact, the duration of successful preclinical research has slightly increased after 2010.

When looking at the relative contribution of different institutional types to the growth of R&D productivity, we found that a relevant fraction of the detected increases are due to better performance of biotechnological companies, in preclinical and clinical research. The rising importance of biotech firms results apparent also when studying the Originator–Developer contractual relationships; in particular, the performance of biotech firms acting as Developers of R&D projects is converging to that of large pharmaceutical companies.

**Table 1:** Average phase-by-phase attrition rates and phase-by-phase share: 2000–2009 (00), 2010–2013 (10), for the three institutional types under study (pharmaceutical and biotechnological companies, non-industrial institutions)

Developer	Attrition rates									
	Preclinical		Phase I		Phase II		Phase III		Registration	
	00	10	00	10	00	10	00	10	00	10
Pharmaceutical	86.80	85.16	62.18	58.91	77.74	78.69	64.33	66.40	46.67	32.52
Biotech	90.25	82.17	59.61	54.15	82.20	77.48	78.15	64.14	35.00	22.54
Non-industrial	96.97	97.07	62.37	59.32	90.00	80.19	82.14	81.25	67.65	0

	Share									
	Preclinical		Phase I		Phase II		Phase III		Registration	
Developer	00	10	00	10	00	10	00	10	00	10
Pharmaceutical	24.16	24.44	43.99	49.15	47.56	48.34	62.26	62.36	76.69	61.19
Biotech	48.60	43.19	44.54	42.51	42.18	44.18	30.55	32.41	16.36	35.32
Non-industrial	27.24	32.37	11.47	8.33	10.26	7.48	7.19	5.24	6.95	3.48

**Table 2:** Regression coefficients for the five cases of Originator–Developer relationship and the two categories of response variables (phase-by-phase transition rates, sales): 1990–1999 (90), 2000–2013 (00)

O$\rightarrow$D	Transition rates									
	Preclinical		Phase I		Phase II		Phase III		Registration	
	90	00	90	00	90	00	90	00	90	00
ni$\rightarrow$ph	− 0.107	− 0.009	− 0.047	0.020	− 0.079	0.024	− 0.067	0.011	− 0.109$^{\dagger }$	0.016
bt$\rightarrow$ph	− 0.044	− 0.029	− 0.005	− 0.048	− 0.009	− 0.042	− 0.080	0.001	− 0.067	0.027
ni$\rightarrow$ni	− 0.291$^{\dagger }$	− 0.275$^{\dagger }$	− 0.232$^{\dagger }$	− 0.212$^{\dagger }$	− 0.188$^{\dagger }$	− 0.155$^{\dagger }$	− 0.148$^{\dagger }$	− 0.094$^{\dagger }$	− 0.145$^{\dagger }$	− 0.047$^{\dagger }$
ni$\rightarrow$bt	− 0.281$^{\dagger }$	− 0.050	− 0.208$^{\dagger }$	− 0.070	− 0.199$^{\dagger }$	− 0.136$^{\dagger }$	− 0.150$^{\dagger }$	− 0.111$^{\dagger }$	− 0.153$^{\dagger }$	− 0.035
bt$\rightarrow$bt	− 0.261$^{\dagger }$	− 0.083$^{\dagger }$	− 0.047	− 0.081$^{\dagger }$	− 0.327$^{\dagger }$	− 0.111$^{\dagger }$	− 0.197$^{\dagger }$	− 0.159	0.024	0.120

O$\rightarrow$D	Sales (log$_{10}$€)		Orphan (%)							
	90	00	90	00						
ni$\rightarrow$ph	0.773$^{\dagger }$	0.123	9.3	9.3						
bt$\rightarrow$ph	0.168	− 0.350$^{\dagger }$	10.3	14.2						
ni$\rightarrow$ni	0.116	− 1.064$^{\dagger }$	11.3	10.1						
ni$\rightarrow$bt	0.123	− 0.598$^{\dagger }$	8.1	13.4						
bt$\rightarrow$bt	0.228	− 0.325$^{\dagger }$	6.7	14.6						

*ni* non-industrial, *ph* pharmaceutical, *bt* biotech.

^†^ Significant at $$p<0.05$$

How much of the improvement in R&D productivity that we documented is structural and how much is transient is an important question for future research. The duration of drug development remains a concern, even though the intensification of the collaboration between firms and regulatory agencies can provide guidance and contribute to positively impact development times (e.g. in Breakthrough Therapy Designation procedures [56]). If the evidences of an increasing productivity will be confirmed, several cohorts of novel therapeutic compounds will reach the market, targeting specific indications and patient groups. A new landscape is emerging, which will be shaped by the coevolution between the progress of the research frontier and the strategies that regulators will implement to deal with new, possible, trade-offs between innovation, access and sustainability.

## Limitations

This study is based on data collected from the R&D Focus dataset, which we have complemented through a significant effort of data integration on patent data, sales figures, and with a classification of institutions and therapeutic indications. Ref. [57] reports missing data issues for ClinicalTrials.gov: despite the fact that institutions are required to insert their results in the database, this has often not been done. R&D Focus mitigates this problem by relying on additional sources such as press releases, conference reports and information gathered directly from companies. Nevertheless, it is not possible to guarantee that the dataset reports all the phase transitions of the described compounds. This is true especially for the Preclinical phase, which typically is not public; 48% of the compounds reporting a Phase I are not associated with any Preclinical phase. These limitations notwithstanding, evidences presented in this paper provide, to our knowledge, the most comprehensive available investigation on recent trends in pharmaceutical R&D. We hope that our results can contribute to show the importance of data provision and integration on all the stages of drug development, with particular reference to detailed information on failures.

## Supplementary information

**Additional file 1.** Additional tables and figures.

## Data Availability

The datasets generated and/or analyzed during the current study are not publicly available due to their proprietary status.

## Footnotes

1 https://www.whocc.no/atc_ddd_index/

2 For instance, https://www.sdrugs.com/?c=atc&n=0

3 A disease is defined as rare if it has a prevalence of $\le$ 200,000 affected individuals in the US. A disease is multifactorial when its causes are represented by the competition of several factors of a different nature, apparently not in direct connection between each other.

4 PCT patents are filed under the Patent Cooperation Treaty. The Patent Cooperation Treaty is an international treaty with more than 150 contracting states, which makes it possible to seek patent protection for an invention simultaneously in a large number of countries. A PCT patent application has the effect of a national patent application in all PCT contracting states.

5 According to the amyloid hypothesis, the main cause of Alzheimer’s disease is the accumulation and deposition of oligomeric or fibrillar amyloid $\beta$ peptide [30].

6 ClinicalTrials.gov [31] is a database of privately and publicly funded clinical studies conducted around the world.
