# Supplementary material for: The endless frontier? The recent increase of R&D productivity in pharmaceuticals
Source: J Transl Med. 2020 Apr 9;18:162. doi: 10.1186/s12967-020-02313-z (PMC7147016; doi:10.1186/s12967-020-02313-z)
Supplement: Supplementary file 1 — Additional file 1. Additional tables and figures. [file 12967_2020_2313_MOESM1_ESM.pdf]

Additional Information

Additional file 1: Table S1 List of ATC1 codes and their names.

ATC1 code	Name
A	Alimentary tract and metabolism
B	Blood and blood forming organs
C	Cardiovascular system
D	Dermatologicals
G	Genito-urinary system and sex hormones
H	Systemic hormonal preparations, excluding sex hormones and insulins
J	Anti-infectives for systemic use
L	Antineoplastic and immunomodulating agents
M	Musculo-skeletal system
N	Nervous system
P	Antiparasitic products, insecticides and repellents
R	Respiratory system
S	Sensory organs

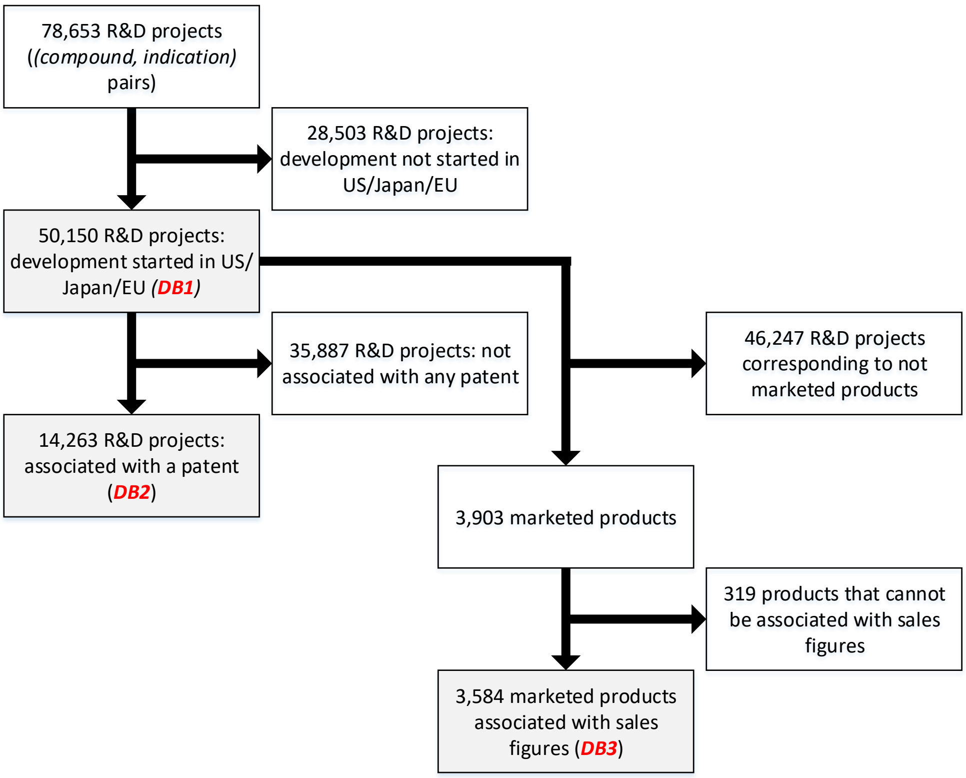

Additional file 1: Fig. S1 Flow chart outlining the selection steps used to build the database used in the experiments. The shaded boxes show the final databases used in the analyses. The database DB1 contains the R&D projects started in US, Japan or EU, and it has been used in the analysis of the attrition rates. The database DB2 contains the subset of the R&D projects covered in DB1, for which an association with the corresponding patents has been established, to study the division of innovative labor between Originators and Developers. The database DB3 contains the subset of the R&D projects covered in DB1 that correspond to marketed products, to analyze the distribution of the projects with respect to probability of success and sales.

Additional file 1: Table S2 Average phase-by-phase attrition rates in 2000-2009 (00), 2010-2013 (10); phase-by-phase share in 2010-2013; p-value of a Wilcoxon test between the distributions of phase transition in the two decades. Projects are divided according to the corresponding first-level ATC class.

	Attrition rates									
	Pr		P_I		P_{II}		P_{III}		Reg	
ATC class	00	10	00	10	00	10	00	10	00	10
A	90.04	89.06	59.33	51.05	76.49	74.03	66.83	65.82	52.54	28.77
B	86.59	83.62	55.42	52.46	83.24	62.50	76.36	83.33	43.24	60.00
C	93.15	93.84	63.58	59.46	84.93	77.44	72.03	71.43	50.00	21.74
D	88.61	87.73	59.20	34.62	83.85	77.35	64.86	73.33	37.25	56.52
G	87.22	82.50	66.67	59.46	83.86	88.60	68.75	71.43	50.75	52.38
H	95.35	90.00	70.37	21.43	91.18	73.91	53.85	88.89	53.85	86.67
J	91.61	91.30	58.93	54.65	84.44	73.17	65.12	55.46	44.14	30.61
L	94.87	91.84	63.39	57.81	84.57	84.44	79.37	76.45	49.69	41.38
M	93.91	90.13	65.86	46.15	81.72	86.77	73.96	78.21	45.16	41.67
N	92.78	91.71	70.18	66.67	82.87	83.81	68.28	77.54	38.61	47.46
P	95.87	98.18	57.14	77.78	92.86	33.33	83.33	100	57.14	50.00
R	91.32	92.67	67.00	64.71	83.64	78.32	89.39	79.25	44.64	58.06
S	91.67	88.40	50.91	36.49	78.49	77.27	66.23	69.09	53.06	61.76

	Share 2010-2013					Wilcoxon p-value				
ATC class	Pr	P_I	P_{II}	P_{III}	Reg	Pr	P_I	P_{II}	P_{III}	Reg
A	8.26	10.04	10.75	16.35	12.99	0.35	$0.004^\dagger$	0.62	0.32	$0.002^\dagger$
B	2.10	2.14	2.82	4.50	1.78	0.52	0.68	$o(10^{-5})^\dagger$	0.39	0.36
C	4.99	3.90	5.26	7.59	4.09	0.85	0.84	$0.02^\dagger$	0.78	$0.01^\dagger$
D	2.95	3.65	5.81	6.26	8.19	0.40	$o(10^{-5})^\dagger$	0.09	0.14	$0.03^\dagger$
G	1.45	2.60	3.66	3.50	7.47	0.16	0.29	0.10	$0.04^\dagger$	0.63
H	0.18	0.49	0.74	0.75	2.67	0.48	$0.003^\dagger$	0.11	0.13	0.08
J	14.75	9.23	7.89	9.92	8.72	0.71	0.19	$0.005^\dagger$	$0.03^\dagger$	0.07
L	36.79	46.77	36.70	24.44	20.64	$o(10^{-10})^\dagger$	$o(10^{-4})^\dagger$	0.92	0.17	0.21
M	4.03	4.56	6.06	6.51	10.68	0.09	$o(10^{-4})^\dagger$	0.39	0.38	0.47
N	16.79	10.11	11.29	11.51	10.50	0.34	0.16	0.94	$0.03^\dagger$	0.28
P	0.99	0.32	0.19	0.33	0.71	0.25	0.56	$0.007^\dagger$	1	1
R	3.45	3.58	4.59	4.42	5.52	0.34	0.38	0.12	0.05	0.21
S	3.27	2.60	4.23	3.92	6.05	0.12	0.06	0.62	0.74	0.67

Additional file 1: Table S3 Advanced therapies (cell and gene therapies), monoclonal antibodies: average attrition rates in 2000-2009 (00), 2010-2013 (10); phase-by-phase share in 2010-2013; p-value of a Wilcoxon test between the distributions of phase transition in the two decades.

	Attrition rates									
	Pr		P_I		P_{II}		P_{III}		Reg	
Category	00	10	00	10	00	10	00	10	00	10
Advanced therapeutics	95.44	86.36	52.17	20.99	89.76	89.90	100	100	50.00	0
Monoclonal antibodies	93.18	87.66	56.76	59.04	77.90	80.81	80.91	69.61	20.51	42.42

	Share 2010-2013					Wilcoxon p-value				
Category	Pr	P_I	P_{II}	P_{III}	Reg	Pr	P_I	P_{II}	P_{III}	Reg
Advanced therapeutics	2.61	4.27	5.17	1.91	0	$0.004^\dagger$	$o(10^{-6})^\dagger$	1	1	/
Monoclonal antibodies	13.92	17.51	15.50	13.93	14.10	$o(10^{-4})^\dagger$	0.53	0.35	0.06	0.05

Additional file 1: Table S4 Alzheimer's disease (AD) and R&D projects referring to the amyloid Alzheimer's disease hypotesis: average phase-by-phase attrition rates in 2000-2009 (00), 2010-2013 (10); phase-by-phase share in 2010-2013; p-value of a Wilcoxon test between the distributions of phase transition in the two decades.

	Attrition rates									
	Pr		P_{I}		P_{II}		P_{III}		Reg	
Category	00	10	00	10	00	10	00	10	00	10
AD	89.77	91.43	73.02	58.82	92.59	80.95	100	100	/	/
AD amyloid	85.71	91.66	59.09	35.29	85.00	83.33	100	100	/	/
	Share 2010-2013					Wilcoxon p-value				
Category	Pr	P_{I}	P_{II}	P_{III}	Reg	Pr	P_{I}	P_{II}	P_{III}	Reg
AD	3.11	1.79	1.10	1.50	0.43	0.79	0.23	0.29	1	/
AD amyloid	1.42	0.90	0.31	0.68	0	0.45	0.25	1	1	/
	Share 2010-2013 (within neurological)					Share 2010-2013 (within AD)				
Category	Pr	P_{I}	P_{II}	P_{III}	Reg	Pr	P_{I}	P_{II}	P_{III}	Reg
AD	14.48	14.35	7.58	9.09	2.08	100	100	100	100	100
AD amyloid	6.62	7.17	2.17	4.13	0	45.71	50.00	28.57	45.45	0

Additional file 1: Table S5 Top-10 diseases by percentage of ongoing clinical studies (source: clinicaltrials.gov). Statistics computed using 33,269 studies whose overall status is "Recruiting", "Active", "Not yet recruiting", "Enrolling by invitation", or "Available". We have excluded the studies with only behavioral interventions and the disease type "Pathological conditions, signs and symptoms". The assignment of a study to a disease type is not exclusive, so that the reported percentages do not sum to 100.

Disease type	Percentage of ongoing clinical studies
Neoplasms	41.59%
Nervous System	20.96%
Cardiovascular	20.69%
Digestive System	17.29%
Immune System	13.29%
Female Urogenital	13.17%
Respiratory Tract	13.03%
Male Urogenital	10.77%
Hemic and Lymphatic	10.37%
Skin and Connective Tissue	10.36%

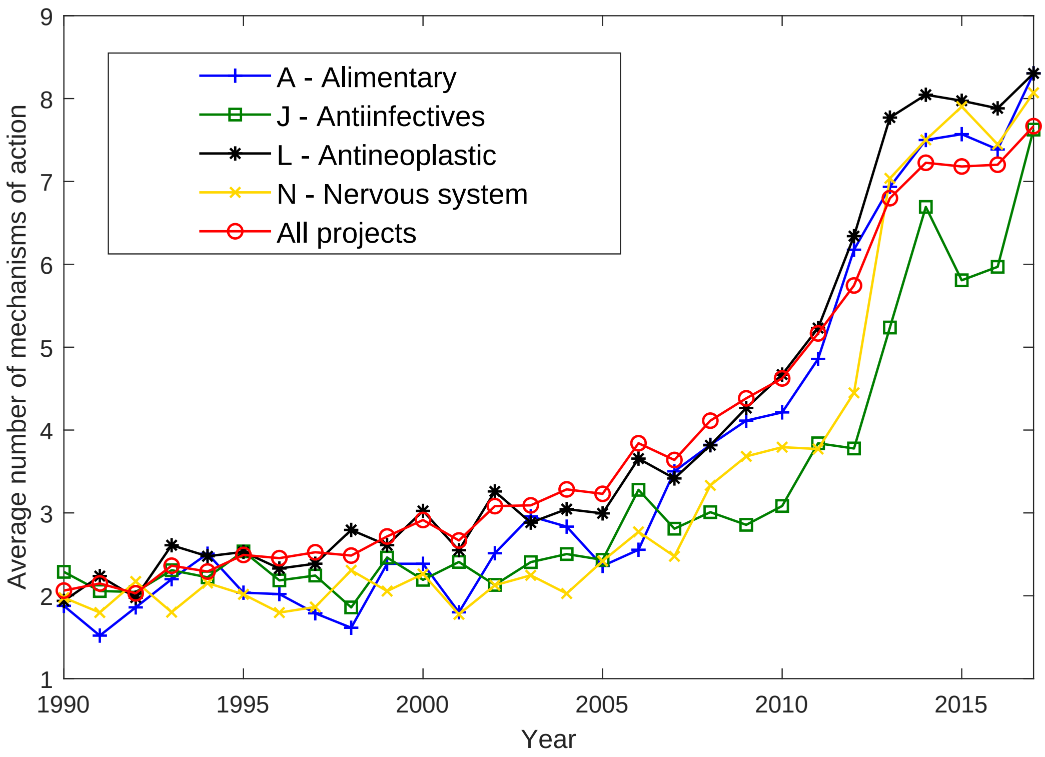

Additional file 1: Fig. S2 Average number of mechanisms of action by ATC1 code. For clarity, we show figures only the four ATC1 classes with the greatest number of Preclinical entries, 2010-2013. The other ATC1 classes follow analogous patterns. The red line shows the trend observed for all the projects reported in Fig. 4a, for comparison.

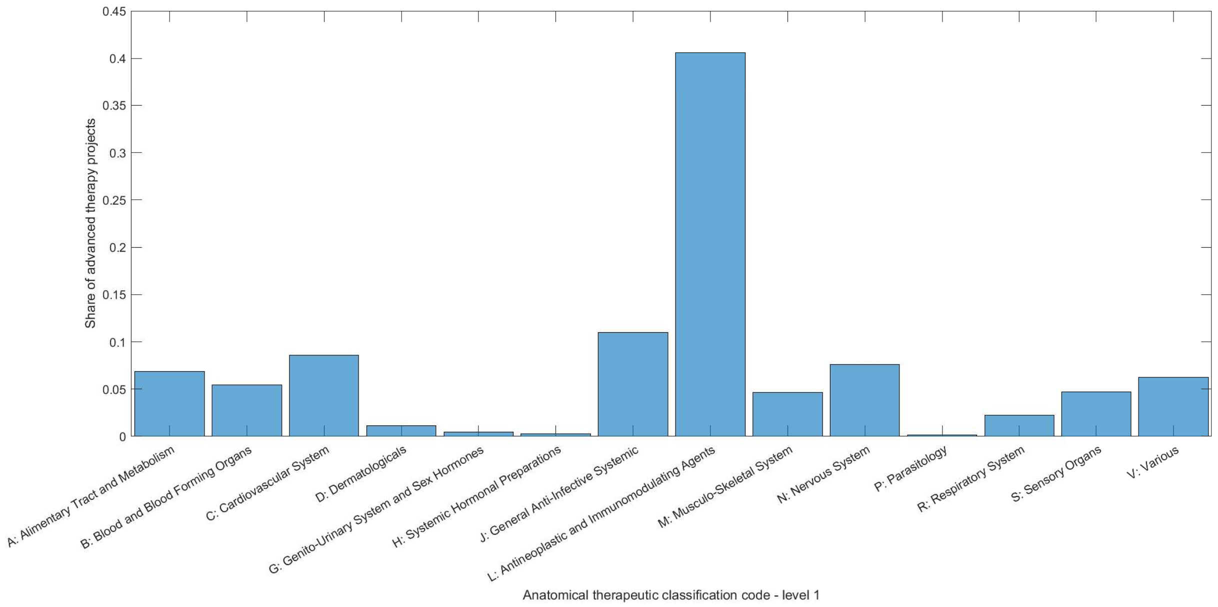

Additional file 1: Fig. S3 Share of R&D projects based on advanced therapies, by ATC code 1.

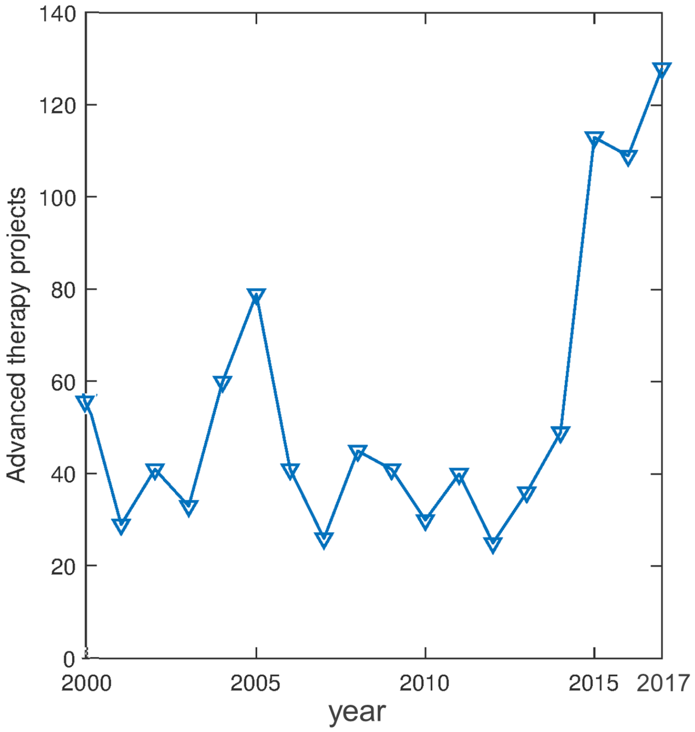

Additional file 1: Fig. S4 Number of R&D projects based on advanced therapies, by starting year.

Additional file 1: Table S6 Average (± standard deviation) yearly phase-by-phase attrition rates in three different time intervals (1990-1999, 2000-2009, 2010-2013), for R&D projects focused on treatment of rare diseases. Rare diseases are identified according to a manual classification performed by a domain expert.

Period	Preclinical	Phase I	Phase II	Phase III	Registration
1990-1999	86.60(± 4.62)%	45.83(± 13.42)%	76.60(± 11.11)%	65.20(± 16.01)%	44.66(± 19.33)%
2000-2009	91.06(± 2.26)%	56.78(± 6.93)%	82.51(± 3.23)%	63.46(± 20.06)%	44.72(± 16.34)%
2010-2013	90.18(± 3.81)%	45.43(± 5.37)%	81.48(± 2.16)%	73.21(± 9.16)%	31.90(± 12.33)%

Additional file 1: Table S7 Average (± standard deviation) yearly phase-by-phase attrition rates in three different time intervals (1990-1999, 2000-2009, 2010-2013), for projects for which we have a classification of the institution of the developer (according to the “pharmaceutical”, “biotech” and “non industrial” classification).

Period	Preclinical	Phase I	Phase II	Phase III	Registration
1990-1999	87.13(± 5.19)%	44.01(± 7.47)%	66.64(± 11.40)%	59.62(± 12.76)%	43.60(± 15.47)%
2000-2009	91.22(± 2.19)%	60.26(± 3.90)%	81.25(± 2.22)%	68.61(± 9.04)%	46.08(± 5.43)%
2010-2013	87.66(± 1.82)%	57.07(± 2.69)%	78.26(± 0.95)%	66.03(± 4.92)%	29.07(± 17.86)%

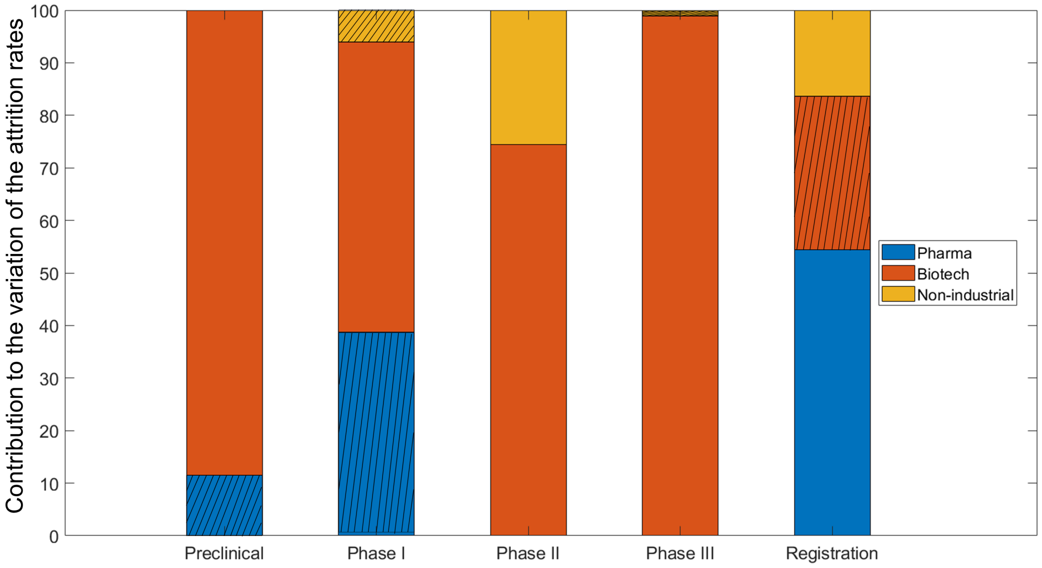

Additional file 1: Fig. S5 Contribution to changes in total phase-by-phase attrition rates from 2000-2009 to 2010-2013 by type of developing institution. The bars expand the attrition rate variation for the focal phase and are divided according to the relative contribution of each institutional type to the observed variation. Contributions related to non-significant changes are barred.

Additional file 1: Table S8 Number of projects (percentage share over the whole period) for each O–D relationship, in 1990-1999 and 2000-2013. *ni*: non-industrial; *ph*: pharmaceutical; *bt*: biotech.

(a) 1990-1999

D O	ph	ni	bt
ph	780 (41.9%)	-	-
ni	56 (0.3%)	393 (21.1%)	104 (5.6%)
bt	129 (6.9%)	-	401 (21.5%)

(b) 2000-2013

D O	ph	ni	bt
ph	1139 (38.0%)	-	-
ni	59 (2.0%)	503 (16.8%)	90 (3.0%)
bt	323 (10.8%)	-	883 (29.5%)

Additional file 1: Table S9 Effect of different Originator-Developer models on project progress and sales.

(a) O: non-industrial; D: pharmaceutical

Variable	OD $type_{90-99}$	N. obs_{90-99}	R^2_{90-99}	OD $type_{00-13}$	N. obs_{00-13}	R^2_{00-13}
Preclinical, transition	-0.107	866	0.299	-0.009	1247	0.322
Phase I, transition	-0.047	866	0.331	0.020	1247	0.378
Phase II, transition	-0.079	866	0.395	0.024	1247	0.358
Phase III, transition	-0.067	866	0.380	0.011	1247	0.366
Registration, transition	-0.109**	866	0.370	0.016	1247	0.387
Sales	0.773**	273	0.550	0.123	450	0.516

(b) O: biotech; D: pharmaceutical

Variable	OD $type_{90-99}$	N. obs_{90-99}	R^2_{90-99}	OD $type_{00-13}$	N. obs_{00-13}	R^2_{00-13}
Preclinical, transition	-0.044	925	0.276	-0.029	1485	0.304
Phase I, transition	-0.005	925	0.323	-0.048*	1485	0.352
Phase II, transition	-0.009	925	0.366	-0.042	1485	0.351
Phase III, transition	-0.080*	925	0.370	0.001	1485	0.366
Registration, transition	-0.067*	925	0.381	0.027	1485	0.383
Sales	0.168	275	0.516	-0.350**	507	0.490

(c) O: non-industrial; D: non-industrial

Variable	OD $type_{90-99}$	N. obs_{90-99}	R^2_{90-99}	OD $type_{00-13}$	N. obs_{00-13}	R^2_{00-13}
Preclinical, transition	-0.291**	1073	0.348	-0.275**	1583	0.418
Phase I, transition	-0.232**	1073	0.385	-0.212**	1583	0.423
Phase II, transition	-0.188**	1073	0.414	-0.155**	1583	0.373
Phase III, transition	-0.148**	1073	0.392	-0.094**	1583	0.360
Registration, transition	-0.145**	1073	0.376	-0.047**	1583	0.381
Sales	0.116	280	0.578	-1.064**	462	0.561

(d) O: non-industrial; D: biotech

Variable	OD $type_{90-99}$	N. obs_{90-99}	R^2_{90-99}	OD $type_{00-13}$	N. obs_{00-13}	R^2_{00-13}
Preclinical, transition	-0.281**	1027	0.336	-0.050	1341	0.331
Phase I, transition	-0.208**	1027	0.363	-0.070*	1341	0.383
Phase II, transition	-0.199**	1027	0.407	-0.136**	1334	0.398
Phase III, transition	-0.150**	1027	0.376	-0.111**	1341	0.371
Registration, transition	-0.153**	1027	0.351	-0.035	1341	0.393
Sales	0.123	280	0.525	-0.598**	452	0.536

(e) O: biotech; D: biotech

Variable	OD $type_{90-99}$	N. obs_{90-99}	R^2_{90-99}	OD $type_{00-13}$	N. obs_{00-13}	R^2_{00-13}
Preclinical, transition	-0.261**	1171	0.344	-0.083**	1947	0.343
Phase I, transition	-0.047	1171	0.305	-0.081**	1947	0.333
Phase II, transition	-0.327**	1171	0.454	-0.111**	1947	0.323
Phase III, transition	-0.197*	1171	0.462	-0.159*	1947	0.474
Registration, transition	0.024	1171	0.524	0.120	1947	0.750
Sales	0.228	298	0.556	-0.325**	586	0.490
